# Chinese version and application of the global health competencies survey for healthcare professionals

**DOI:** 10.3389/fpubh.2025.1624826

**Published:** 2025-10-01

**Authors:** Xiaoxi Li, Junjie Jia, Jingjing Hu, Xuqi He, Meiqiong Zheng

**Affiliations:** ^1^Department of Hematology, Henan Provincial People's Hospital, Zhengzhou, China; ^2^Department of Respiratory, Henan Provincial People's Hospital, Zhengzhou, China; ^3^Department of Ophthalmology, Henan Provincial People's Hospital, Zhengzhou, China

**Keywords:** global health competencies, healthcare professionals, survey validation, cultural adaptation, Chinese healthcare, health inequities

## Abstract

**Objective:**

This study aimed to introduce and validate the global health competencies survey (GHCS) in the Chinese healthcare context, addressing the need for a comprehensive tool to assess global health competencies among diverse healthcare professionals.

**Methods:**

The GHCS underwent meticulous translation and cultural adaptation, engaging 150 healthcare professionals from various disciplines between 1^st^ June and 10^th^ December, 2023. The study employed a rigorous methodology involving instrument development, translation, data collection, and statistical analysis. Face and content validity, factor analysis, and internal consistency were assessed to validate the survey.

**Results:**

The translated GHCS demonstrated robust reliability (Cronbach’s alpha = 0.841) and validity. The survey identified competencies, showcased temporal trends, and informed targeted interventions. No floor or ceiling effects were observed, except for one variable (racial/ethnic disparities).

**Conclusion:**

The adapted and validated GHCS emerges as a valuable tool for assessing global health competencies among Chinese healthcare professionals. Implications for research use include identifying knowledge gaps, facilitating program improvements, and contributing to reduced health inequities. Despite limitations, such as the absence of criterion validation and Mandarin administration, the GHCS offers a foundation for further research and program enhancements in China.

## 1 Introduction

As global health challenges become increasingly complex—ranging from emerging infectious diseases to climate-related health crises—the need for healthcare professionals with cross-cultural awareness, global health literacy, and systems-thinking capabilities is more urgent than ever. These global health competencies are essential for meaningful engagement in international health cooperation, disease prevention, humanitarian aid, and health equity advancement.

China has emerged as a key global health actor, playing an expanding role in health diplomacy, medical aid, and international health workforce deployment. Through programs such as dispatching medical teams to Africa, supporting the World Health Organization, and responding to public health emergencies abroad (e.g., COVID-19 aid missions), Chinese healthcare professionals are increasingly required to work in diverse, cross-border contexts (1).

Furthermore, China’s commitment to global health cooperation is institutionalized within its Belt and Road Initiative (BRI) (2), particularly the Health Silk Road, which promotes bilateral and multilateral health collaboration, knowledge exchange, and capacity-building with countries along the BRI corridor. These developments signal a strategic national shift toward integrating global health competencies into healthcare education and workforce planning (3).

However, despite this strategic alignment, there remains a notable gap in standardized tools to assess and develop these competencies within the Chinese context. Existing instruments may not adequately reflect the cultural, linguistic, and systemic realities of Chinese healthcare practice.

Therefore, adapting and validating a culturally appropriate tool—such as the Global Health Competencies Survey (GHCS)—is critical for ensuring that China’s healthcare workforce is equipped to meet both domestic and international health challenges in a globally interconnected era. The GHCS, originally developed and validated by Mirella et al. (4), is a self-assessment tool designed to measure a broad spectrum of knowledge and interest in global health and health equity, global health skills, and needs in global health education (1–3, 5).

The GHCS was selected for this study due to its structured, multi-domain approach, strong psychometric properties, and track record of successful cross-cultural adaptation in multiple international contexts. Compared to more conceptual frameworks like the Consortium of Universities for Global Health (CUGH) competencies, which emphasize educational outcomes and curriculum design, the GHCS provides a practical, itemized instrument for measuring individual-level competencies quantitatively. While the CUGH framework is valuable for guiding program development, it lacks a standardized survey format validated for psychometric evaluation.

Given the GHCS’s modular structure, its adaptability, and its coverage of both knowledge and attitude-based domains, it offers superior structural suitability for localization in the Chinese healthcare context. The choice of GHCS thus ensures methodological rigor, content relevance, and compatibility with the study’s goals of assessment, comparison, and future application in training and workforce development. Modern healthcare demands not only clinical excellence but also proficiency in navigating global health determinants, cross-cultural interactions, and transnational health systems. Accordingly, global health competencies have emerged as essential tools in equipping healthcare workers to meet these multidimensional demands.

The practice of healthcare has transcended national boundaries. Healthcare professionals are now frequently engaged in international collaborations, responses to global disease outbreaks, and efforts to reduce health disparities across diverse populations (5–7). These evolving responsibilities call for a strategic reevaluation of the competencies required for modern healthcare practice—competencies that extend beyond traditional clinical skills to encompass global health literacy, cultural adaptability, and system-level thinking.

The Global Health Competencies Survey (GHCS) was developed to assess such capabilities in healthcare professionals (8). Although the instrument has been validated and is widely applied in Western countries, its suitability for non-Western contexts—such as China—remains insufficiently explored (12). This represents a significant gap, given China’s expanding role in global health diplomacy, medical aid, and international health collaboration (3).

This study aims to fill this gap by validating the GHCS for use in the Chinese healthcare context, with three main objectives: (1) Translation and Cultural Adaptation: To ensure linguistic clarity and contextual appropriateness for Chinese healthcare professionals. (2) Psychometric Evaluation: To rigorously assess the reliability and validity of the GHCS within a Chinese cultural and professional framework. (3) Applied Relevance: To explore the practical utility of the GHCS in real-world Chinese healthcare settings for identifying training needs and informing targeted capacity-building initiatives. Ultimately, this study supports the broader dialog on global health preparedness and provides an actionable instrument to guide the development of globally competent healthcare professionals in China.

## 2 Methodology

### 2.1 Participants

This study included a total of 150 healthcare professionals from three Grade A tertiary hospitals across China. In the Chinese healthcare system, Grade A tertiary hospitals represent the highest-ranking public hospitals, characterized by their comprehensive clinical services, advanced medical technology, teaching responsibilities, and active engagement in medical research and international collaboration. These institutions are typically located in major cities and serve as referral centers for complex or critical cases.

A purposive sampling strategy was employed to ensure diverse representation across key healthcare roles, including physicians, nurses, and allied health professionals (e.g., therapists, pharmacists). Grade A tertiary hospitals were selected specifically due to their broad spectrum of specialties, institutional capacity, and greater likelihood of exposure to global health activities, such as international partnerships, cross-border patient care, or participation in multinational training initiatives. These hospitals also tend to employ staff with higher educational attainment and greater access to professional development resources, making them particularly suitable for evaluating global health competencies.

Other hospital grades, such as secondary or lower-tier tertiary hospitals, were not included in this phase of the study to ensure environmental consistency and maximize institutional comparability. Including hospitals of varying levels could have introduced confounding variables related to resource availability, professional exposure, and education infrastructure. Future studies will be necessary to examine the generalizability of findings across other healthcare settings.

Inclusion criteria required participants to be active-duty healthcare professionals (physicians, nurses, or allied health staff) with a minimum of one year of clinical work experience. The one-year threshold was set to ensure that participants had achieved basic professional integration, including familiarity with workplace protocols, interprofessional communication, and patient care routines. While more experienced professionals could offer deeper insight, limiting the sample to individuals with over 5 or 10 years of experience would have significantly reduced sample accessibility and excluded early-career professionals, who also play a critical role in healthcare delivery and training pipelines. The chosen threshold thus ensured both data quality and sample representativeness.

Participants were invited through hospital administrative offices and department leads. From June 1 to August 1, 2023, a pilot survey was conducted with 30 healthcare professionals from one hospital to test the clarity and timing of the questionnaire. The average completion time was 10 min, and no major revisions were needed. The formal data collection phase occurred from August 3 to December 10, 2023, using a secure online platform. The participants, actively engaged in healthcare roles for at least one year, provided signed informed consent, ensuring commitment and willingness to participate in the study.

Exclusion criteria included healthcare workers not currently engaged in clinical duties (e.g., on long-term leave, study abroad, or administrative secondment), as well as interns and students, whose limited clinical experience may not sufficiently reflect applied global health competencies.

The sample size of 150 participants was calculated using a commonly accepted ratio of 5:1 (participants per questionnaire item) based on the 30-item GHCS (22 global health competencies items and 8 demographic questions), consistent with previous psychometric validation studies (7–9).

### 2.2 Instrumentation

The primary measurement instrument used in this study was the Global Health Competencies Survey (GHCS), originally developed and validated by Mirella et al. (4), as a structured self-assessment tool designed to evaluate a broad range of global health competencies (9). The scale comprises three subscales (46 items in total): 1) Global health knowledge/interest (17 items, 3-point Likert scale), with higher scores indicating greater confidence and deeper understanding; 2) Cross-cultural nursing competence (14 items, 5-point Likert scale), where elevated scores reflect stronger self-perceived skills; 3) Global health education needs (15 items, 6-point Likert scale), with higher ratings denoting greater perceived necessity. The overall scale demonstrated good reliability (Cronbach’s α = 0.862). Demographic background questions were additionally included.

The original instrument was developed in English and has demonstrated sound psychometric properties across different cultural settings. For this study, the GHCS was translated and culturally adapted into Chinese using a forward–backward translation protocol, followed by expert review and pilot testing to ensure linguistic equivalence and cultural appropriateness in the Chinese healthcare context.

To ensure transparency, replicability, and future applicability, the full Chinese version of the GHCS instrument used in this study has been provided as a supplementary file (Supplementary material 1).

### 2.3 Translation and data collection

The GHCS was translated and culturally adapted into Chinese in accordance with established cross-cultural adaptation guidelines. The adaptation process involved forward translation by two bilingual healthcare professionals fluent in English and Mandarin, followed by backward translation by two independent translators with no prior exposure to the original instrument. Discrepancies were resolved through consensus meetings with the research team to ensure conceptual equivalence rather than literal translation.

To enhance semantic clarity and cultural relevance, two senior editors with expertise in public health and health communication conducted semantic proofreading. The revised version was then reviewed by the core research team and pilot-tested with 30 healthcare professionals to confirm item clarity and content validity.

The final Chinese version of the GHCS was distributed via the Wenjuanxing platform (www.wjx.cn)—a widely used, secure Chinese online survey tool that supports mobile and desktop access, automated response collection, and time-stamped data exports. Participants received the survey link through institutional communication channels, along with an electronic informed consent form embedded at the beginning of the survey. Reminder notifications were sent at 2 and 4 weeks to enhance response rates. Online surveys were chosen for their efficiency, faster response rates, and broader accessibility (9, 11).

### 2.4 Validity

Validity refers to the degree to which an instrument accurately measures the construct it is intended to assess (12). In this study, we employed a multi-pronged approach to evaluate the validity of the Chinese version of the GHCS, combining face validity, content validity, and item-level performance analysis to ensure methodological rigor.

Face validity, though considered a subjective and lower-level form of evidence, is particularly relevant and commonly employed in early phases of cross-cultural instrument adaptation (13). It was used here as a preliminary step to assess whether the translated items were understandable, culturally appropriate, and perceived by the target population to reflect global health competencies. This assessment involved structured feedback from 30 healthcare professionals during the pilot phase.

To strengthen the instrument’s validity, we also conducted a content validity evaluation through an expert panel comprising global health educators, clinicians, and public health researchers. Experts reviewed the instrument for relevance, clarity, and comprehensiveness, offering item-specific recommendations that informed iterative revisions.

In addition, we examined floor and ceiling effects (14) across all items to evaluate whether the instrument was capable of detecting variability in responses. Floor effects occur when a large proportion of respondents choose the lowest possible score, indicating insensitivity at the lower end of the scale. Conversely, ceiling effects occur when respondents disproportionately select the highest score. A well-calibrated tool should demonstrate low floor and ceiling effects, allowing discrimination across a range of competency levels (15). In our study, most domains exhibited minimal floor or ceiling effects, supporting the content-related validity of the GHCS in this context.

Taken together, this triangulated approach—including face validity, expert-informed content validation, and item performance analysis—provides a more robust foundation for claiming the instrument’s appropriateness for use in the Chinese healthcare setting, beyond relying on face validity alone.

### 2.5 Reliability and internal consistency

Internal consistency allows an evaluation of questionnaire reliability by gauging how effectively items within a specific domain complement each other (13). This assessment relies on a single administration of the survey (16). To evaluate the internal consistency of our multi-item instrument, we employed Cronbach’s alpha. Items exhibiting item-total correlation values below 0.2 were eliminated. We deemed alpha values exceeding 0.70 as the benchmark for satisfactory questionnaire reliability (17).

### 2.6 Data analysis

The quantitative data obtained from the GHCS responses underwent comprehensive analysis using SPSS 22.0. Descriptive statistics illuminated the demographic characteristics of the participants, while inferential statistics, examined the underlying constructs of global health competencies.

## 3 Results

### 3.1 Demographic characteristics

The study enrolled a total of 150 healthcare professionals from three Grade A tertiary hospitals in China. The participant pool reflected a professionally diverse cohort, including physicians (*n* = 50), nurses (*n* = 60), and allied health professionals (*n* = 40) such as rehabilitation therapists, pharmacists, and medical technologists. This multidisciplinary composition was intended to ensure broad representation across different functional roles in healthcare delivery.

All participants had a minimum of one year of clinical experience, with nearly one-third (32%) having 2–5 years of professional service, and 20.67% reporting over 5 years of experience. This distribution allowed inclusion of both early-career and mid-career professionals, balancing perspectives from newly integrated staff and more seasoned practitioners. Table 1 presents the full demographic and baseline characteristics of the participants.

**Table 1:** Demographic and baseline characteristics of participants.

Characteristics	N	%
**Age (mean, years)**	–	32.54
**Sex**		
Male	61	40.67
Female	89	59.33
**Marital status**		
Married	90	60
Unmarried	60	40
**Years of work experience**		
1–2 years	21	14
2–5 years	48	32
>5 years	81	54
**Department/discipline**		
Internal medicine	39	26
Surgery	32	21.33
Infectious diseases	18	12
Emergency/critical care	20	13.33
Public health/preventive medicine	14	9.33
Pediatrics	10	6.67
Other	17	11.34

All participants were recruited from three Grade A tertiary hospitals, but some worked in affiliated community or specialty branches administratively linked to those tertiary centers. These subunits may be classified as non–Grade A facilities, hence the discrepancy in the “Hospital Level” variable.

### 3.2 Face and content validity

Face and content validity of the Chinese GHCS were confirmed following expert review and item analysis. Key findings included the following:

Modifications were made to 6 items, including adjustments to terminology (e.g., replacing abstract policy terms with practical clinical equivalents) and clarification of response stems.
No items were removed, but 2 items were reordered to improve logical flow (e.g., grouping similar themes such as health disparity and social determinants).
Response format (Likert scale) was retained based on expert consensus, as it was deemed culturally appropriate and consistent with the original instrument’s structure.
Expert feedback also led to minor revisions in item phrasing to ensure cultural sensitivity and relevance without altering the core constructs.

**Figure 1:**
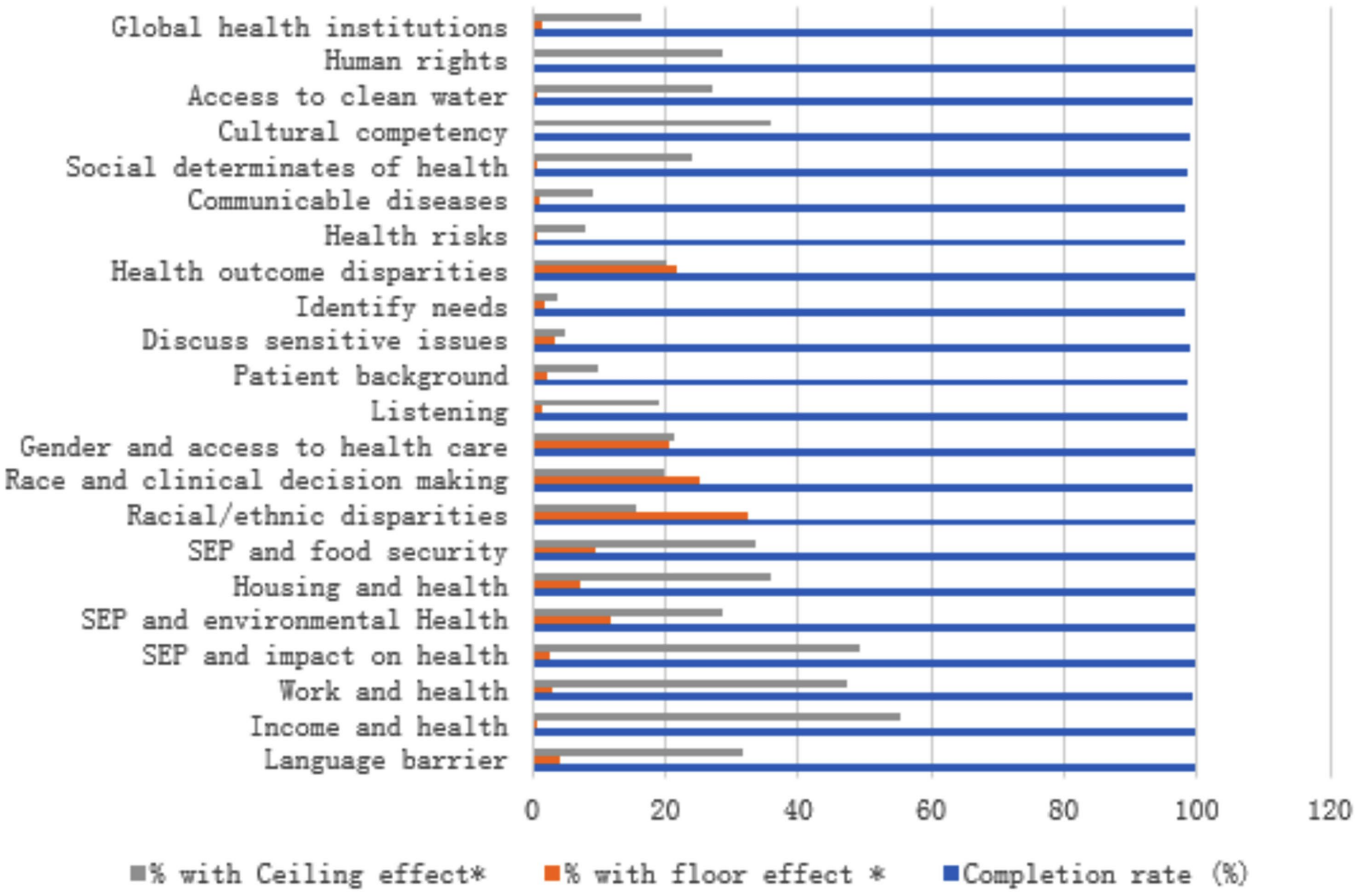
Ceiling and floor effect for each domain. This figure displays the distribution of responses across the different domains of the GHCS, highlighting the absence of ceiling and floor effects. Each domain’s results show a balanced spread of scores, indicating that the instrument effectively captures the full range of global health competencies without bias toward extreme values.

While there is no universally agreed-upon cutoff point for ceiling and floor effects (18), many studies suggest that these effects may occur when more than one-third of the total population achieves either the best or worst scores, respectively (>33%) (19, 20). In our study, one variable (racial/ethic disparities) exhibited a floor effect. For the overall rating score, no participants exhibited floor or ceiling effects (Table 2; Figure 1).

**Table 2:** Ceiling and floor effect for each domain.

Items	Completion rate (%)	% With floor effect	% With ceiling effect
Language barrier	99.7	4.2	31.5
Income and health	99.9	0.8	55.2
Work and health	99.4	3	47.3
Socioeconomic position and impact on health	99.8	2.5	49.1
Socioeconomic position and environmental health	99.9	11.8	28.6
Housing and health	99.6	7.2	35.9
Socioeconomic position and food security	99.7	9.5	33.7
Racial/ethnic disparities	99.9	32.5	15.7
Race and clinical decision making	99.4	25.3	19.8
Gender and access to health care	99.8	20.7	21.4
Listening	98.7	1.5	18.9
Patient background	98.5	2.2	9.8
Discuss sensitive issues	98.8	3.5	4.9
Identify needs	98.2	1.8	3.9
Health outcome disparities	99.9	21.7	20.3
Health risks	98.3	0.7	7.8
Communicable diseases	98.2	1.2	9.2
Social determinants of health	98.5	0.9	24.1
Cultural competency	98.9	0.3	35.8
Access to clean water	99.2	0.8	27.2
Human rights	99.7	0.4	28.7
Global health institutions	99.4	1.5	16.5

### 3.3 Internal consistency

Internal consistency of the Chinese version of the GHC survey was calculated using Cronbach’s alpha coefficient, which provides information regarding the strength of inter-item correlation. The reliability analysis of the 22 items obtained a Cronbach’s alpha coefficient of 0.841 (Table 3).

**Table 3:** Reliability analysis of the 22 items (Cronbach’s alpha = 0.841).

Items	Obs	Item-test correlation	Item-rest correlation	Cronbach’s alpha if item deleted
Language barrier	150	0.4521	0.3825	0.8573
Income and health	148	0.5543	0.4891	0.8537
Work and health	149	0.5127	0.4453	0.8559
Socioeconomic position and impact on health	150	0.5612	0.4968	0.8542
Socioeconomic position and environmental health	148	0.5891	0.5294	0.8548
Housing and health	150	0.6065	0.5471	0.8543
Socioeconomic position and food security	149	0.6034	0.5432	0.8545
Health outcome disparities	148	0.5936	0.5256	0.8551
Social determinants of health	150	0.5512	0.4856	0.8567
Cultural competency	149	0.4423	0.3678	0.8596
Access to clean water	148	0.5334	0.4659	0.8562
Human rights	150	0.5156	0.4428	0.8571
Global health institutions	149	0.4889	0.4173	0.8582
Listening	150	0.2998	0.2187	0.8648
Patient background	149	0.3256	0.2423	0.8637
Discuss sensitive issues	148	0.3479	0.2675	0.8629
Identify needs	150	0.3221	0.2408	0.8636
Racial/ethnic disparities	149	0.5498	0.4837	0.8568
Race and clinical decision making	150	0.5952	0.5356	0.8559
Gender and access to health care	149	0.6031	0.5436	0.8553
Health risks	148	0.4482	0.3756	0.8597
Communicable diseases	150	0.4534	0.3802	0.8594

### 3.4 Scree plot of GHCS factor structure

**Figure 2:**
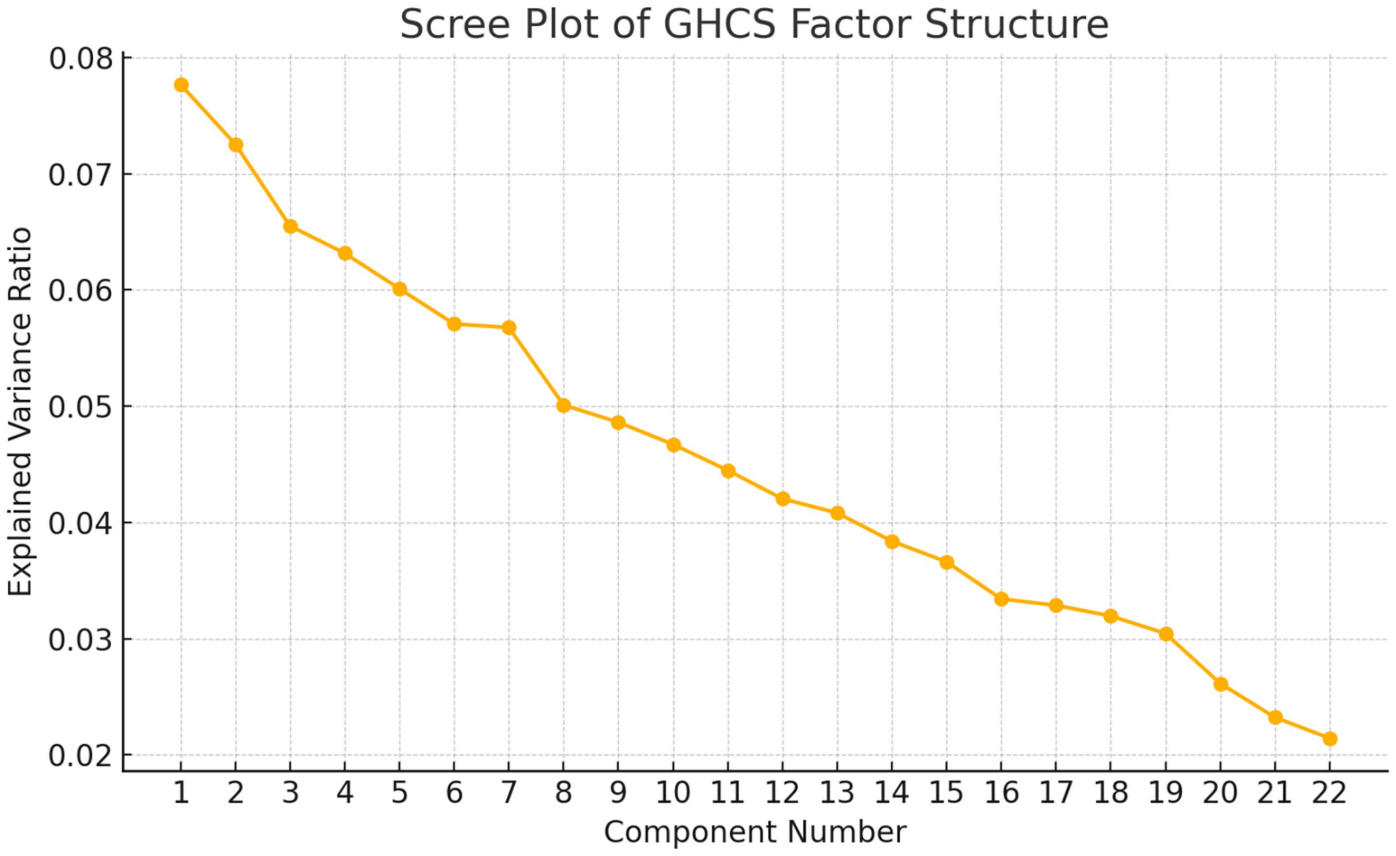
Scree plot of GHCS factor structure.

Figure 2 is the scree plot displaying the explained variance of each principal component from the factor analysis (via PCA). This visualization helps determine the optimal number of factors to retain—commonly where the “elbow” occurs, indicating diminishing returns in explained variance.

### 3.5 Factor loading heatmap

**Figure 3:**
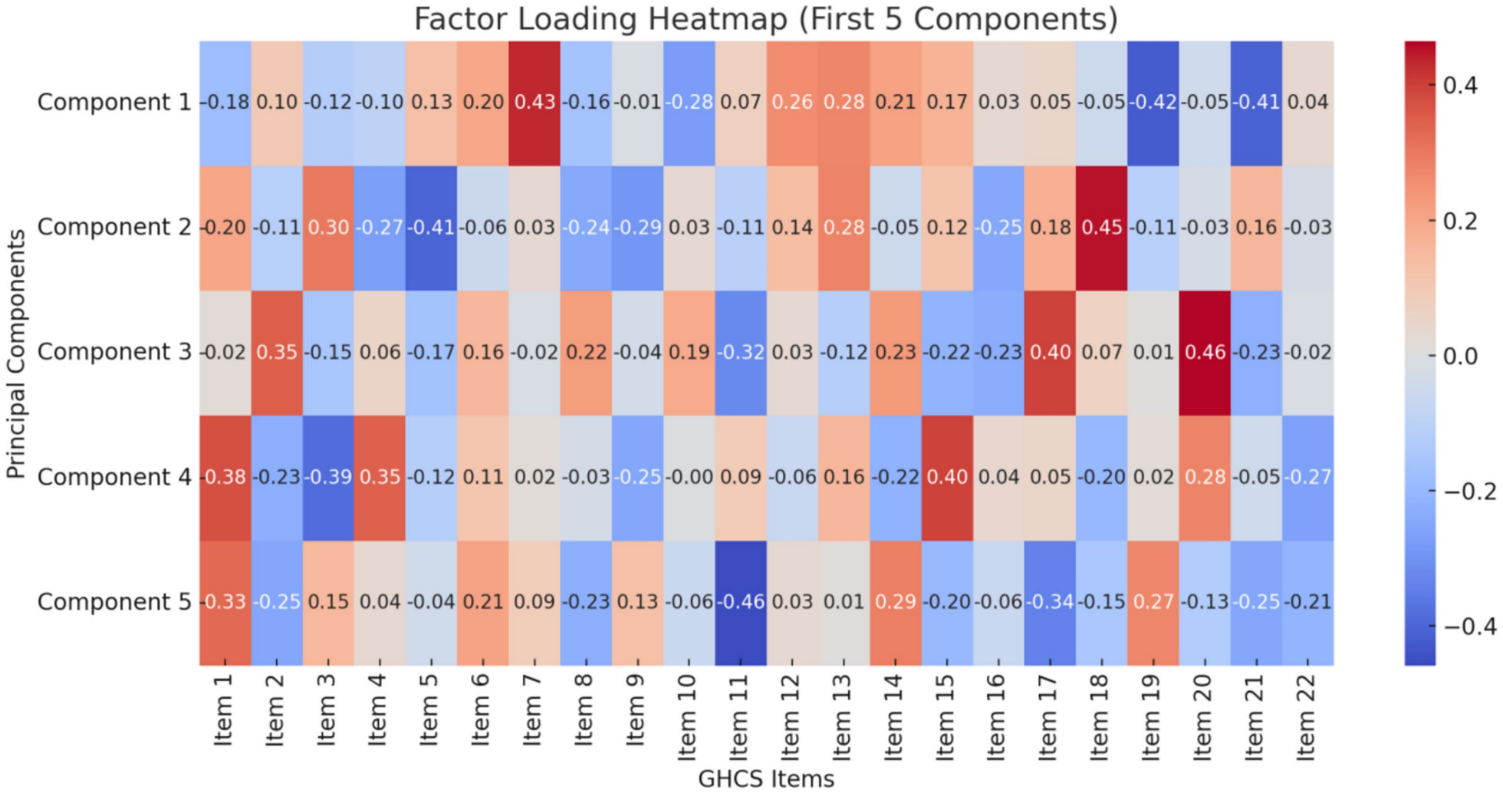
Factor loading heatmap.

Figure 3 is the factor loading heatmap for the first 5 components of the GHCS. This visualization shows how strongly each item correlates with each principal component, offering insight into the underlying factor structure.

## 4 Discussion

By validating a standardized and internationally recognized tool, this research supports strategic capacity building, equipping educators, institutions, and policymakers with an evidence-based framework to guide global health training initiatives. Moreover, the adapted GHCS serves as a bridge between global standards and local relevance, enhancing China’s readiness for active and effective participation in global health engagement.

This study successfully validated the GHCS for use among Chinese healthcare professionals, demonstrating strong psychometric performance in terms of internal consistency, face and content validity, and a well-defined factor structure. The rigorous translation and cultural adaptation process—guided by expert panels and pre-survey testing—ensured both linguistic accuracy and contextual relevance, critical for capturing the nuances of global health competencies in the Chinese healthcare context. The Cronbach’s alpha coefficient of 0.841 means that it proved reliable, sharing the same psychometric properties with prior studies, which had shown the GHCS to be valid for Western populations (21). The absence of significant ceiling and floor effects in most domains suggests the tool effectively captures a broad spectrum of competencies without biasing results toward low or high performers. Our scree plot and factor loading heatmap further revealed a stable underlying factor structure, supporting the construct validity of the GHCS in this new setting.

Notably, Chinese healthcare professionals scored particularly high in domains such as cultural competency, knowledge of global health institutions, and social determinants of health. These results echo the growing national emphasis on international health collaboration, health diplomacy, and global health education initiatives in China (22, 23). Compared to previous validations, our study achieved stronger psychometric performance, potentially due to a more comprehensive cultural adaptation process, including iterative expert review and semantic refinement, as recommended by Ramada-Rodilla et al. (24).

Interestingly, the results suggest Chinese professionals may exhibit stronger global health awareness in certain areas than their Western counterparts, likely reflecting evolving educational reforms and China’s increasing presence in global health affairs. This also highlights the importance of contextual adaptation—not merely translating but tailoring instruments to local realities to enhance their validity and utility.

The GHCS holds valuable potential across multiple sectors. For educational institutions, universities and training centers can employ the GHCS to assess baseline global health competencies and tailor curricula accordingly. Strengths in high-performing domains may be reinforced, while areas with lower scores—such as understanding racial and ethnic disparities—can prompt the development of targeted modules or case-based learning. For healthcare administrators, hospitals and health bureaus may use the GHCS as a needs-assessment tool to identify gaps in global health competencies within their workforce. These insights can support the integration of relevant content into continuing professional development, particularly for staff engaged in cross-border or multicultural care. For policymakers, government agencies and health commissions can leverage aggregate GHCS data to shape national strategies for global health workforce development and to benchmark progress toward broader goals in health diplomacy and international cooperation.

This study presents several notable strengths. The GHCS underwent rigorous cultural adaptation and validation, incorporating expert feedback and pre-survey testing to ensure contextual relevance. It demonstrated strong internal consistency, along with clear evidence of both content and construct validity. Additionally, visual analyses—such as scree plots and heatmaps—helped elucidate the factor structure, further supporting the instrument’s dimensional robustness.

### 4.1 Study strengths and implications

This study represents a pioneering effort in adapting and validating the GHCS for use in the Chinese context. It provides a culturally tailored and psychometrically robust tool for assessing global health competencies among Chinese healthcare professionals. Key areas of strength identified among participants included cultural competency, familiarity with global health institutions, and understanding of social determinants of health—findings that align with China’s increasing role in global health collaboration and education. The absence of significant ceiling and floor effects further supports the tool’s appropriateness for capturing a wide spectrum of competencies.

### 4.2 Practical applications for policy and education

The validated GHCS holds significant promise for educators, administrators, and policymakers. It offers an evidence-based instrument to inform the design, evaluation, and improvement of global health training programs within China’s health system. Its application can support the strategic development of global health curricula, facilitate benchmarking of institutional training efforts, and enhance the global readiness of China’s health workforce. By aligning international standards with local realities, the GHCS contributes to strengthening national capacity for global health engagement.

### 4.3 Limitations of the study

Sample size and representativeness: The sample comprised 150 participants from three Grade A tertiary hospitals, which may not fully capture the diversity of China’s healthcare workforce, especially those working in rural or lower-tier institutions. Although specific quantitative data on international work or training exposure was not collected, the selection of leading hospitals—known for academic exchange, foreign patient services, and institutional collaborations—suggests that a portion of participants may have had indirect exposure to global health contexts.

Cross-sectional design: This limits our ability to evaluate changes in competencies over time or in response to specific interventions.

Self-report bias: As with all self-assessment instruments, responses may be influenced by social desirability, especially in culturally sensitive or aspirational domains like global awareness and equity.

### 4.4 Recommendations for future research

Future research should address these limitations through several approaches. Expanding sampling to include a broader range of provinces, urban and rural settings, and various healthcare institution levels would enhance generalizability. Longitudinal designs are needed to assess how competencies develop over time and in response to training or real-world global health engagement. Incorporating educational intervention trials would allow testing the effectiveness of GHCS-informed curricula. Additionally, triangulating self-reported data with peer evaluations, supervisor ratings, or observed performance metrics could help mitigate response bias and enrich validity.

This work lays a strong foundation for ongoing global health education research and practice in China. The GHCS provides a reliable and contextually adapted tool to guide curriculum development, workforce planning, and institutional benchmarking. By supporting longitudinal and interventional studies, this research contributes to building a globally competent health workforce prepared to address emerging international health challenges.

## 5 Conclusion

This study provides the first comprehensive validation of the GHCS for use among Chinese healthcare professionals, confirming its reliability, validity, and cultural relevance. The findings highlight strong internal consistency, robust content and face validity, and an interpretable factor structure aligned with global health competency domains.

Key areas of strength among Chinese professionals included cultural competency, awareness of global health institutions, and understanding of social determinants of health—reflecting China’s growing engagement in global health education and international collaboration. The absence of ceiling and floor effects in most domains confirms the tool’s suitability for capturing the full range of competencies.

The validated GHCS offers a practical, evidence-based instrument for use by educators, administrators, and policymakers to evaluate, monitor, and enhance global health training in China. Its application can inform targeted educational interventions, support national workforce strategies, and contribute to China’s preparedness for global health engagement.

This work lays a strong foundation for future longitudinal and interventional research, advancing both academic understanding and practical capacity building in global health education across China’s healthcare system.

## Data Availability

The original contributions presented in the study are included in the article/Supplementary material, further inquiries can be directed to the corresponding author/s.
