# Supplementary material for: Chinese version and application of the global health competencies survey for healthcare professionals
Source: Front Public Health. 2025 Oct 1;13:1624826. doi: 10.3389/fpubh.2025.1624826 (PMC12521418; doi:10.3389/fpubh.2025.1624826)
Supplement: Supplementary file 1 [file Data_Sheet_1.docx]

# Supplementary Material 1

全球卫生能力调查问卷（Global Health Competencies Survey, GHCS）– 中文版

说明：请根据您对以下陈述的同意程度进行评分。
使用以下5点李克特量表作答：
1 = 非常不同意，2 = 不同意，3 = 一般，4 = 同意，5 = 非常同意

1. 1. 我能够识别并克服语言障碍以有效沟通。
2. 2. 我理解收入水平如何影响健康状况。
3. 3. 我了解工作条件对健康的影响。
4. 4. 我理解社会经济地位对健康的总体影响。
5. 5. 我能分析环境因素如何影响社会经济地位人群的健康。
6. 6. 我认识到住房条件对健康的潜在影响。
7. 7. 我了解食品安全和营养如何影响不同人群的健康。
8. 8. 我理解种族/民族差异对健康公平的影响。
9. 9. 我了解种族在临床决策中的潜在作用。
10. 10. 我理解性别如何影响获取健康服务的机会。
11. 11. 我能倾听并尊重患者的观点和背景。
12. 12. 我在提供护理服务时会考虑患者的文化背景。
13. 13. 我能够在敏感议题上与患者进行有效沟通。
14. 14. 我能够识别患者的具体健康需求。
15. 15. 我认识到不同群体在健康结果方面存在差异。
16. 16. 我了解特定人群所面临的健康风险。
17. 17. 我了解传染病的传播机制与防控策略。
18. 18. 我理解社会决定因素在全球健康中的作用。
19. 19. 我具备与不同文化人群有效互动的文化能力。
20. 20. 我理解获取清洁水源的重要性及其健康影响。
21. 21. 我认识到人权在健康服务提供中的核心地位。
22. 22. 我了解世界卫生组织及其他全球卫生机构的功能。
