# Supplementary material for: Chinese version and application of the global health competencies survey for healthcare professionals
Source: Front Public Health. 2025 Oct 1;13:1624826. doi: 10.3389/fpubh.2025.1624826 (PMC12521418; doi:10.3389/fpubh.2025.1624826)
Supplement: Supplementary file 2 [file Data_Sheet_2.docx]

# Appendix A: Summary of GHCS Item Revisions Based on Expert Panel Feedback

This appendix details the modifications made to the Chinese version of the Global Health Competencies Survey (GHCS) based on expert feedback during face and content validity review.

| Item Number | Original Wording | Revised Wording | Type of Change |
| --- | --- | --- | --- |
| Item 3 | 我了解工作条件对健康的影响。 | 我能够识别工作环境中可能影响健康的具体因素（如长时间轮班、接触有害物质等）。 | Clarified scope with contextual examples |
| Item 8 | 我理解种族/民族差异对健康公平的影响。 | 我认识到不同人种或族群在获取健康服务时可能面临的障碍。 | Rephrased for sensitivity and clarity |
| Item 14 | 我能够识别患者的具体健康需求。 | 我能够根据患者背景评估其潜在健康需求。 | Expanded clinical context |
| Item 16 | 我了解特定人群所面临的健康风险。 | 我能识别某些人群（如老年人、低收入者）在特定环境下的健康风险。 | Contextualization with population examples |
| Item 20 | 我理解获取清洁水源的重要性及其健康影响。 | 我了解在资源有限地区获取清洁饮水的挑战及其对公共健康的影响。 | Revised for global relevance |
| Item 22 | 我了解世界卫生组织及其他全球卫生机构的功能。 | 我能够简要说明世界卫生组织（WHO）在全球卫生中的作用及主要任务。 | Increased specificity |
